# Miscellany

**Published:** 1902-05

**Authors:** 


### Miscellany.

---

TO BEND A CROWN-POST WITHOUT STRAIN ON THE CROWN.—Grasp the post with a pair of crown contouring pliers. The convex jaw of the pliers forces a portion of the post into the concave jaw, and thus bends it without danger to the porcelain crown.—*Pacific Medical Journal.*

---

BELL-SHAPED CROWN, TO BE APPLIED WITHOUT TRIMMING THE TOOTH.—The following is a concise description of an original and clever device of Dr. McDonaugh's.

In the molar to be crowned, measure the distance from the highest cusp to the largest part of the swell of the crown, then from this point to the neck, then the circumference at the largest part of the swell. (The crown is made in two sections, an upper and a lower, corresponding to the above measurements.) Make the lower section of pure gold and to fit only at the swell of the crown. The upper section is made of 22-carat gold and is accurately adapted to the tooth. Place in position on the tooth. On the lingual side pinch the gold, making it fit accurately at the neck. This is comparatively easy, on account of the lower part of the crown being made of pure gold. With curved shears cut away the part pinched up. Remove the crown. Unite the crack made by cutting off the pinched part by placing over it and soldering to it a fairly wide and thick piece of platinum; two pieces of square post-metal will answer the purpose. File this to a dovetail. Make a female part of the dovetail with overlapping flanges of pure gold, tightly adapted to the lingual surface of the crown. Saw a slit up the platinum dovetail, and the crown is ready for setting.—*Dominion Dental Journal.*

---

A New Method for closing Superficial Incised Wounds.—Dr. Arthur G. Bretz (*Medical and Surgical Monitor*, December, 1900) believes the method very simple, and describes it as follows:

Given a wound on the forehead, for instance, after cleansing and preparing it in the usual way, dry the adjacent surface thoroughly and then apply a piece of adhesive plaster on either side of the wound, the size of the plaster and the distance from the edge of the wound to be determined by the length and character of the same. However, it should be of sufficient width to give ample area for adhesion, which should be not less than one-fourth of an inch and not nearer the wound than one-fourth of an inch. Raise the inner edges of the adhesive strips and insert interrupted sutures through them instead of through the skin, draw together, and tie. This coaptates the edges of the wound even better than stitches through the skin. The wound is then dressed in the usual way.

First, it prevents the painful process of inserting stitches, of which all patients have such a dread.

Secondly, it does away with the possibility of stitch-hole abscess and the trouble caused by particles of sutures being left in the wound on removing the stitches.

Thirdly, it prevents the stitch-marks, which always add to the unsightliness of the scar.

Fourthly, in cases of wounds inflicted by a blunt instrument, which caused bruised tissue immediately surrounding the wound, there are no stitches to tear out the friable tissue.

There is no puckering between the stitches; the first stitches coaptate the edges, and the others make the closure permanent. There are many other advantages besides those enumerated above. —*Therapeutic Gazette.*

